# Radiation dose optimization for photon-counting CT coronary artery calcium scoring for different patient sizes: a dynamic phantom study

**DOI:** 10.1007/s00330-023-09434-1

**Published:** 2023-02-02

**Authors:** Magdalena M. Dobrolinska, Niels R. van der Werf, Judith van der Bie, Joël de Groen, Marcel Dijkshoorn, Ronald Booij, Ricardo P. J. Budde, Marcel J. W. Greuter, Marcel van Straten

**Affiliations:** 1 Department of Radiology, University Medical Center Groningen, University of Groningen, Groningen, The Netherlands; 2 Division of Cardiology and Structural Heart Diseases, Medical University of Silesiain , Katowice, Katowice, Poland; 3 Department of Radiology & Nuclear Medicine, Erasmus University Medical Center, Rotterdam, The Netherlands; 4 Department of Robotics and Mechatronics, University of Twente, Enschede, The Netherlands

**Keywords:** X-ray computed tomography, Calcium, Coronary vessels, Imaging phantoms

## Abstract

**Purpose:**

To systematically assess the radiation dose reduction potential of coronary artery calcium (CAC) assessments with photon-counting computed tomography (PCCT) by changing the tube potential for different patient sizes with a dynamic phantom.

**Methods:**

A hollow artery, containing three calcifications of different densities, was translated at velocities corresponding to 0, < 60, 60–75, and > 75 beats per minute within an anthropomorphic phantom. Extension rings were used to simulate average- and large -sized patients. PCCT scans were made with the reference clinical protocol (tube potential of 120 kilovolt (kV)), and with 70, 90, Sn100, Sn140, and 140 kV at identical image quality levels. All acquisitions were reconstructed at a virtual monoenergetic energy level of 70 keV. For each calcification, Agatston scores and contrast-to-noise ratios (CNR) were determined, and compared to the reference with Wilcoxon signed-rank tests, with *p* < 0.05 indicating significant differences.

**Results:**

A decrease in radiation dose (22%) was achieved at Sn100 kV for the average-sized phantom. For the large phantom, Sn100 and Sn140 kV resulted in a decrease in radiation doses of 19% and 3%, respectively. Irrespective of CAC density, Sn100 and 140 kVp did not result in significantly different CNR. Only at Sn100 kV were there no significant differences in Agatston scores for all CAC densities, heart rates, and phantom sizes.

**Conclusion:**

PCCT at tube voltage of 100 kV with added tin filtration and reconstructed at 70 keV enables a ≥ 19% dose reduction compared to 120 kV, independent of phantom size, CAC density, and heart rate.

**Key Points:**

*• Photon-counting CT allows for reduced radiation dose acquisitions (up to 19%) for coronary calcium assessment by reducing tube voltage while reconstructing at a normal monoE level of 70 keV*.

*• Tube voltage reduction is possible for medium and large patient sizes, without affecting the Agatston score outcome.*

## Introduction

An important step in clinical radiology is the advent of photon-counting CT [1–6]. This new technology employs smaller detector elements than conventional CT. These smaller detector elements directly convert individual incoming photons into electrical signals and register these individually in predefined energy bins, as opposed to indirect conversion which includes first the transition of photon energy to visible light after which this is converted to an electrical signal. Moreover, only integration over multiple photons was feasible with conventional CT, where PCCT allows for the detection of single photons. PCCT, therefore, has several benefits: the availability of spectral data for each CT exam, as well as increased spatial resolution, and a reduced impact of electronic noise [1, 3, 7].

As shown in ROBINSCA trial, coronary artery calcium (CAC) can be used as a screening tool to guide preventive treatment in asymptomatic individuals [8]. According to CAC’s screening potential, the reduction of radiation dose of CAC scan would be especially of interest. Thanks to the availability of spectral data for each CT scan, an important use case is related to CAC quantification as this could result in reduced radiation dose [2, 9–14]. Quantification of CAC is clinically performed according to the Agatston methodology [15]. This methodology dictates the use of 120-kilovolt (kV) tube voltage for all patients, independent of patient size. As a result, a single threshold (130 Hounsfield units (HU)) can be used for CAC discrimination. However, the most efficient way to decrease radiation dose for CT is to decrease the tube voltage to patient-size–specific values [16–21]. This is especially interesting considering the increase of CT CAC assessments and its related increase in cumulative radiation dose [22]. For conventional CT, a change in tube voltage results in a change in CT numbers of CAC. To be able to use these images for CAC assessment, several solutions have been proposed to overcome any difference in CAC quantification. These solutions include specific calcium-aware reconstruction kernels or adjusted CAC scoring thresholds [16–21]. For PCCT, however, it is possible to maintain CT numbers while scanning at different tube potentials by reconstructing the same virtual monoenergetic image (VMI) level. Specifically for CAC assessments, this means data acquisition at a patient-size––specific tube potential, and reconstructing at a VMI level of 70 kiloelectron volt (keV) which corresponds to the conventional 120-kV reconstructions [12, 14, 23].

For a clinical dual-source PCCT, it was previously shown with the aid of a dynamic phantom that a tube potential of 90 kV allowed for reproducible Agatston scores for large phantom sizes in comparison with standard 120-kV acquisitions [12]. The impact on a medium-sized phantom remains unknown. This is of particular interest for an updated version of this same PCCT system, which allows for a larger variety of tube potentials (70, 90, Sn100, 120, Sn140, and 140), eligible for VMI reconstructions of 70 keV.

The aim of the current study is, therefore, to systematically assess the radiation dose reduction potential of CAC assessments at all available tube voltages with a PCCT system for different patient sizes with a dynamic phantom.

## Methods

**Fig. 1:**
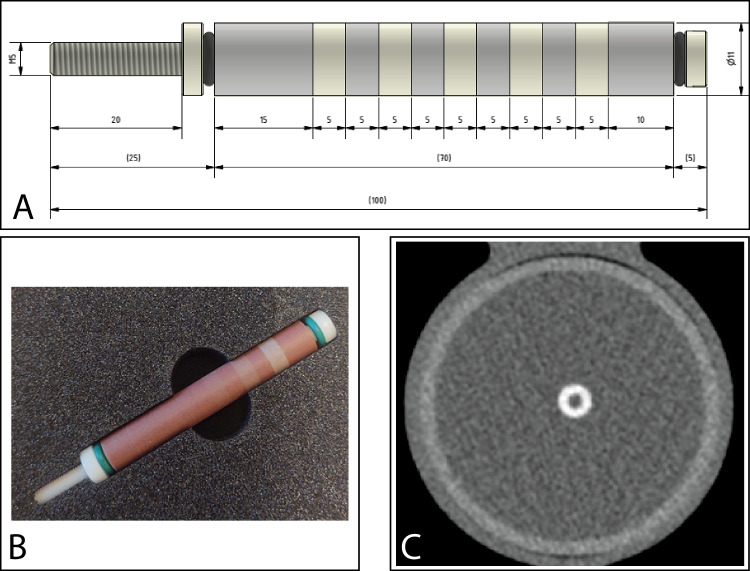
Representations of the hollow artificial artery: **A** schematic overview with dimensions in millimeters, with the solid water (gray) and hydroxyapatite calcifications (yellow) indicated, (**B**) photograph, and (**C**) a 70*-*keV reconstructed cross-sectional image of the medium*-*density calcification (window width/window level at 750/90 Hounsfield units)

A hollow artificial artery (inner diameter 0.5 cm, outer diameter 1.1 cm) was positioned within a water compartment at the center of an anthropomorphic thorax phantom (QRM-thorax, PTW) (Fig. 1). The artery was made of solid water (0 HU) and contained three hollow cylindrical hydroxyapatite (HA) calcifications of identical dimensions (inner diameter 0.5 cm, outer diameter 1.1 cm, length 0.5 cm), but different densities. The HA densities were 200, 400, and 800 mg/cc, designated as low, medium, and high density, respectively. Both the water compartment and the inner lumen of the artery were filled with water. The artery was translated by a robotic arm (Sim2D, PTW) perpendicular to the z-axis at velocities of 0, 10, 20, and 30 mm/s, approximately equivalent to the mean in vivo motion of coronary arteries during the scan phase at heart rates of 0, < 60, 60–75, and > 75 beats per minute (bpm), respectively [24–26]. The electrocardiogram output of the robotic arm was used to ensure data acquisition during linear motion of the robot, without any turning points. To simulate average and large patient sizes, medium and large fat-tissue equivalent extension rings (M and L extension ring, PTW) were positioned around the thorax phantom increasing the outer dimensions to 350 mm × 200 mm and 400 mm × 300 mm, respectively [27].

First, the dynamic phantom was scanned on a dual-source PCCT (NAEOTOM Alpha, Siemens Healthineers) with the routinely used clinical reference protocol: tube potential 120 kV; axial scan technique; automatic tube current modulation (ATCM) with image quality level 16 (CareIQ, Siemens Healthineers), reconstruction technique filtered-back-projection (FBP or QIR off); field-of-view 220 mm; matrix 512 × 512; slice thickness/increment at 3/1.5 mm; reconstruction kernel Qr36. Second, additional acquisitions were performed for both phantom sizes at 70, 90, Sn100, Sn140, and 140 kV, at the same image quality level of 16 using ATCM that aims at keeping image noise constant while changing kV. All scans were reconstructed at a VMI level of 70 keV. Each acquisition was repeated five times, with manual repositioning of the setup between each scan (2 mm translation, 2° rotation), to introduce inter-scan variability.

For each calcification, Agatston scores were automatically determined with a previously validated Python script, using CT vendor-specific CAC scoring parameters [28]. As all scans were reconstructed at a VMI level of 70 keV, the conventional Agatston threshold of 130 HU was applied. All voxels exceeding this threshold were considered to be CAC.

For each calcification density, the contrast-to-noise ratio (CNR) was determined to assess potential differences between each acquisition and the reference. With this, the influence of tube potential on signal intensity and image noise was assessed. The CNR was calculated as:

$$CNR=\frac{\left|{Mean}_{CAC}-{Mean}_{Background}\right|}{{Standard deviation}_{Background}}$$

where Mean_CAC_ was the mean CT number of all voxels exceeding the CAC scoring threshold, and Mean_Background_ and Standard deviation_Background_ the mean CT number and standard deviation of the CT numbers within a region-of-interest of 450 voxels (15 × 30) in the water compartment.

Statistical analyses were performed using SPSS version 27 (SPSS, IBM). For each tube potential, differences in CNR or Agatston score with the reference protocol values were assessed with the use of Wilcoxon signed-rank tests. *p* values below 0.05 were considered to indicate a statistically significant difference.

## Results

### Radiation dose

**Fig. 2:**
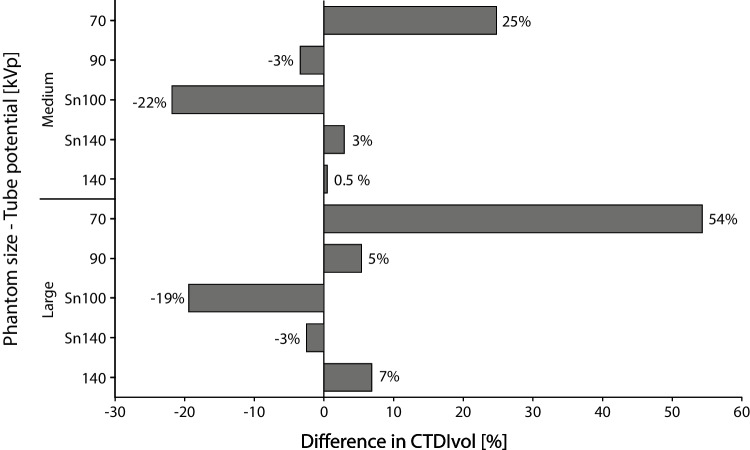
For each phantom size, volumetric CT dose index (CTDI_vol_) differences for all tube potentials with respect to the reference acquisition at 120 kV

The effective tube current time products, based on the ATCM level 16, are indicated in Table 1. Reference volumetric CT dose index (CTDI_vol_) values, retrieved from the radiation dose structured report, at 120 kV, were 2.06 and 2.78 mGy for the medium and large phantom sizes, respectively. For the medium phantom, the largest decrease in radiation dose with respect to the reference at 120 kV was achieved for Sn100-kV acquisitions, at 22% (Fig. 2). Acquisitions at 90 kV also reduced the radiation dose, while all other tube potentials increased radiation dose for medium-sized patients. For the large phantom, Sn100- and Sn140-kV acquisitions resulted in a decrease in radiation doses of 19% and 3%, respectively.

**Table 1:** Effective tube current time products for different tube potentials and phantom sizes

Tube potential (kV)	Effective tube current time product (mAs)	
	Medium phantom	Large phantom
120	13	18
70	90	151
90	29	43
Sn100	95	132
Sn140	29	37
140	9	13

#### CNR

In comparison with the reference CNR of the stationary CAC for the large phantom, reference CNR were higher for the medium-sized phantom for all CAC densities (Table 2). For the low-density CAC, significantly different (*p* < 0.05) CNRs were shown for the large phantom at 70 kV, the medium phantom at 90 kV, and the medium phantom at Sn140 kV. For the medium-density CAC, only the medium-sized phantom at Sn140 kV resulted in a significantly different (*p* = 0.043) CNR in comparison with the reference. For the high-density CAC, significantly different CNR were not shown for the used tube potentials and phantom sizes.

**Table 2:** Median (total range) contrast-to-noise ratios for all tube potentials, phantom sizes*,* and coronary artery calcium densities at rest. Bold values indicate a significant difference from the respective phantom size and density calcification at a tube potential of 120 kV

Tube potential (kV)	Phantom size	Low density		Medium density		High density	
		CNR	*p*	CNR	*p*	CNR	*p*
120	Medium	9.2 (8.7−9.6)	Ref	14.5 (14.1−15.9)	Ref	24.8 (23.2−27.6)	Ref
	Large	7.0 (6.2−8.0)	Ref	10.9 (9.9−12.5)	Ref	18.4 (16.2−21.1)	Ref
70	Medium	8.8 (8.1−9.2)	0.138	14.6 (13.4−15.0)	0.225	23.7 (23.0−25.2)	0.500
	Large	8.0 (7.6−9.3)	**0.043**	12.3 (11.8−13.8)	0.080	20.1 (19.2−22.4)	0.225
90	Medium	8.5 (7.6−9.0)	**0.043**	13.6 (12.1−15.0)	0.080	22.6 (21.6−25.0)	0.080
	Large	6.9 (6.0−8.0)	0.500	10.7 (9.5−12.8)	0.345	17.9 (15.6−20.6)	0.225
Sn100	Medium	8.6 (7.1−9.8)	0.225	14.5 (11.7−15.4)	0.225	24.1 (20.6−25.8)	0.138
	Large	7.1 (6.7−8.1)	0.345	11.2 (10.7−12.8)	0.345	18.1 (16.9−20.7)	0.893
Sn140	Medium	8.0 (7.1−8.3)	**0.043**	12.8 (11.6−13.9)	**0.043**	21.3 (19.9−23.8)	0.080
	Large	6.2 (5.8−6.5)	0.080	9.8 (9.6−10.3)	0.080	15.8 (15.7−16.8)	0.080
140	Medium	9.1 (8.5−9.4)	0.686	14.3 (13.5−15.2)	0.343	24.6 (22.4−25.7)	0.686
	Large	7.3 (6.9−8.3)	0.225	11.6 (10.7−13.1)	0.138	19.1 (18.0−21.9)	0.138

### Agatston scores

**Fig. 3:**
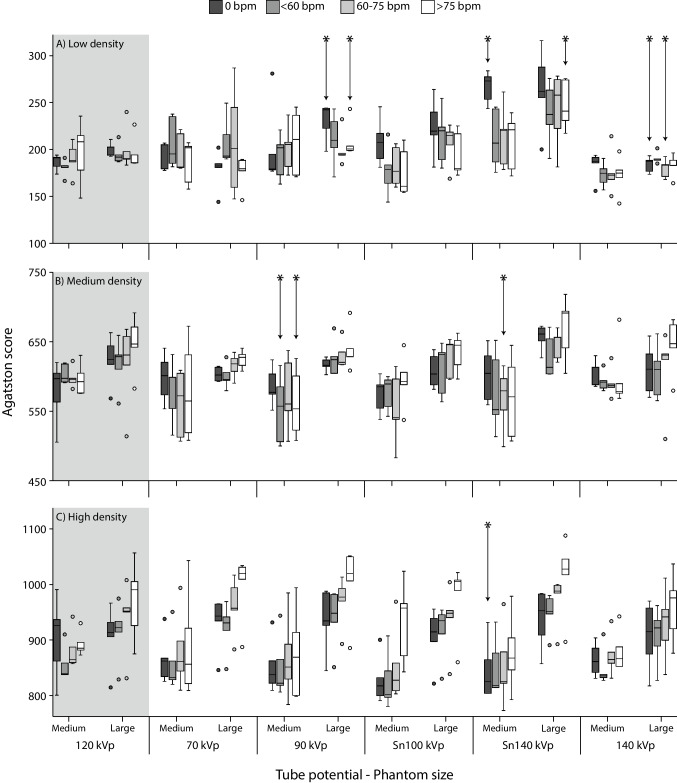
Agatston scores for low (**A**), medium (**B**), and high (**C**) density coronary artery calcifications (CAC). For each CAC density, box and whisker plots are presented for all tube potentials, phantom sizes, and four heart rates. The reference acquisition (120 kV) is indicated by the gray area. For each phantom size and heart rate, significantly different Agatston scores are indicated with an asterisk

For both the low- and medium-density CAC, Agatston scores at 70 and Sn100 kV acquisitions did not show significant differences (*p* > 0.05) with the reference for all phantom sizes and heart rates (Fig. 3). For the medium-density CAC, this also applied to the 140-kV acquisitions. For the high-density CAC, only Sn140-kV acquisitions of the static CAC within the medium-sized phantom resulted in a significantly different (*p* = 0.043) Agatston score in comparison with the reference. For 70, 90, Sn100, and 140 kV, Agatston scores were not shown to differ significantly from the reference for the high-density CAC.

## Discussion

This study has identified that employing a tube potential of Sn100 kV results in a radiation dose reduction of ≥ 19% without showing significantly different CNR or Agatston scores, independent of phantom size, CAC density, or heart rate. At other tube potentials, significant differences in CNR with the reference did not always result in significantly different Agatston scores.

Our study is the first to assess the potential of tube voltage–independent calcium scoring for dynamic CAC in different patient sizes. In a static phantom study, Mergen et al found accurate Agatston scores (≤ 5% percentage deviation with conventional CT) for 90- and Sn100-kV acquisitions independent of phantom size [29]. Their study, however, did not assess interscan variability, with only 1 scan per acquisition and reconstruction combination. For our study, with 5 repeated measurements, significant differences in Agatston scores were shown for at least one CAC density for acquisitions at 90 kV. Finally, as a lower standard ATCM level was used, our study indicated smaller levels of radiation dose reduction in comparison with the reference clinical protocol at 120 kV. Van der Werf et al previously found a radiation dose reduction potential of 57% for dynamic medium- and high-density CAC when acquiring data at 90 kV with reduced tube current levels [12]. In our current study, which employed differently shaped calcifications, however, 90 kV resulted in significantly different Agatston scores in comparison with the reference for low- and medium-density CAC. For Sn100 kV, significant differences with the reference were not indicated, also not for low-density CAC. In our current study, we found for the large phantom slightly different radiation dose levels in comparison with the previous study [12]. We hypothesize that this difference is the result of using an updated software version of the PCCT system (VA50), differences in pitch values, and the limited reproducibility of ATCM. Nevertheless, in both studies, the radiation dose reduction was highest at Sn100 kV when using ATCM at a constant IQ level.

The ATCM used image noise to define the target IQ level. Alternatively, one might target at the CNR of CAC. Although this makes sense physically, it has drawbacks as well. It would require a new way of calcium scoring because HU numbers of CAC might change and image noise might increase resulting in falsely detected CAC. In this study, priority was given to the applicability of the conventional Agatston scoring methodology.

According to clinical guidelines, CAC should be taken into consideration when planning lipid-lowering therapy [30, 31]. Moreover, follow-up CAC scans can be applied as a monitoring of atherosclerotic disease [32]. Taking all things together, the increasing importance of CAC scanning will lead to a growing number of patients who need a CAC scan. Therefore, we should aim for clinical protocols which enable scan acquisition at a lower radiation dose. Importantly, as depicted on Fig. 2, a tube potential of Sn100 kV decreased the radiation dose in both patient sizes. In addition, as there was no difference in Agatston score between the proposed acquisition parameters and reference scan, we believe it might be applied in daily clinical practice after clinical validation.

This study has limitations. First, an anthropomorphic phantom with a hollow artificial artery was used for the current study. The outer dimensions of this phantom were needed to enable the drilling of the internal lumen. These outer dimensions (11-mm diameter) are large compared to the dimensions of in vivo calcifications. Therefore, these results need further validation in a patient study. Next, only phantom data was included in the current analysis. Clinical validation of our results is needed in advance of widespread clinical use.

To conclude, PCCT allows for a radiation dose reduction of ≥ 19% by merely changing the tube potential to Sn100 kV, without changing image quality or Agatston score outcome.

## Abbreviations

ATCM: Automatic tube current modulation
CAC: Coronary artery calcium
CNR: Contrast-to-noise ratio
CTDI_vol_: Volumetric CT dose index
HA: Hydroxyapatite
HU: Hounsfield units
keV: Kiloelectron volt
kV: Kilovolt
PCCT: Photon-counting CT
VMI: Virtual monoenergetic image

## Declarations

### Guarantor

The scientific guarantor of this publication is Marcel van Straten.

### Statistics and biometry

No complex statistical methods were necessary for this paper.

### Methodology

• prospective

• experimental

• performed at one institution
